# Upregulation of nuclear factor E2-related factor 2 (Nrf2) represses the replication of herpes simplex virus type 1

**DOI:** 10.1186/s12985-021-01733-7

**Published:** 2022-01-31

**Authors:** Li Zhang, Jiao Wang, Zhan Wang, Ying Li, Hui Wang, Hongtu Liu

**Affiliations:** 1 National Institute for Viral Disease Control and Prevention, Chinese Center for Disease Control and Prevention, Beijing, 102206 China; 2 Center for Biosafety Mega-Science, Chinese Academy of Science, Wuhan, 430071 Hubei China; 3 Chinese Center for Disease Control and Prevention-Wuhan Institute of Virology, Chinese Academy of Sciences Joint Research Center for Emerging Infectious Diseases and Biosafety, Wuhan, 430071 Hubei China; 4 Key Laboratory for Medical Virology Ministry of Health and Family Planning Commission, Beijing, 102206 China

**Keywords:** Nuclear factor E2-related factor 2 (Nrf2), Herpes simplex virus type 1 (HSV-1), Oxidative stress, Heme oxygenase-1 (HO-1), NAD(P)H quinone oxidoreductase 1 (NQO1)

## Abstract

**Background:**

Nuclear factor E2-related factor 2 (Nrf2) is an important transcription factor which plays a pivotal role in detoxifying reactive oxygen species (ROS) and has been more recently shown to regulate inflammatory and antiviral responses. However, the role of Nrf2 in Herpes Simplex Virus type 1 (HSV-1) infection is still unclear. In this study, the interaction between the Nrf2 and HSV-1 replication was investigated.

**Methods:**

The levels of oxidative stress was monitored by using 8-hydroxy-2'-deoxyguanosine (8-OHdG) ELISA kits, and the dynamic changes of Nrf2-antioxidant response element (Nrf2-ARE) pathway were detected by Western Blot. The effect of Nrf2-ARE pathway on the regulation of HSV-1 proliferation was analyzed by Western Blot, Real-Time PCR and TCID_50_ assay.

**Results:**

HSV-1 infection induced oxidative stress. Nrf2 was activated, accompanied by the increase of its down-stream antioxidant enzyme heme oxygenase-1 (HO-1) and NAD(P)H quinone oxidoreductase 1 (NQO1) in the early stage of HSV-1 infection. The proliferation of HSV-1 was inhibited by overexpression of Nrf2 or treatment with its activator tert-Butylhydroquinone (tBHQ). On the contrary, silencing of Nrf2 promotes virus replication. HO-1 is involved in the regulation of IFN response, leading to efficient anti-HSV-1 effects.

**Conclusion:**

Our observations indicate that the Nrf2-ARE pathway activates a passive defensive response in the early stage of HSV-1 infection. Targeting the Nrf2 pathway demonstrates the potential for combating HSV-1 infection.

## Introduction

Herpes Simplex Virus type 1 (HSV-1) is a prevalent and important human pathogen with a worldwide seroprevalence of more than 80% in adults. Despite considerable effort, there is still no cure and no vaccine for HSV-1 [1]. Oxidative stress-induced by viral infection is one of the major pathogenic mechanisms and may contribute to inflammatory responses and tissue injury by disrupting the balance of intracellular redox-cycling [2]. Reactive oxygen species (ROS) generated by HSV-1 infection have critical effects on viral replication [3] and sensitizes the cells to apoptosis [4].

Nuclear factor E2-related factor 2 (Nrf2) is a central transcription factor regulating cellular adaptive response to oxidative stress. Under non-stressed conditions, Nrf2 is expressed at low level because of its interaction with Kelch-like ECH-associated protein 1 (Keap1), resulting in the ubiquitination and degradation of Nrf2, while, upon the stress signal, Nrf2 dissociated from Keap1 and translocated to the nucleus, then binds to DNA sequences termed antioxidant response element (ARE) and subsequently initiates the transcription of antioxidant genes including heme oxygenase-1 (HO-1), NAD(P)H quinone oxidoreductase 1 (NQO1), which protects cells against the detrimental effects of different stresses [2].

Regulation of the Nrf2-antioxidant defense systems is involved in viral susceptibility, virus-associated inflammation, and immune clearance. Several agonists, including tert-Butylhydroquinone (tBHQ), D,L-Sulforaphane (SFN), and dimethyl fumarate (DMF), target Nrf2 and exhibit antiviral activity in Rabbit Hemorrhagic Disease Virus(RHDV), Spring Viraemia of Carp Virus (SVCV), and SARS-CoV2 infection [5–7]. However, the effect of HSV-1 infection on the Nrf2 pathway is still unclear, and the role of Nrf2 in HSV-1 infection needs to be further clarified.

In this study, we aimed to investigate the role of Nrf2 in HSV-1 infected HEp-2 cells. Our data showed that HSV-1 infection induced oxidative stress, Nrf2 was activated in the early stage of viral infection. Upregulation of Nrf2 effectively inhibited the proliferation of HSV-1. However, silencing of Nrf2 promotes virus replication. Nrf2-ARE pathway is involved in the regulation of oxidative stress and inflammatory upon HSV-1 infection. Activation of the Nrf2-dependent antioxidative pathway might represent a feasible therapeutic approach against HSV-1 infection.

## Materials and methods

### Cells, virus, plasmid, and chemicals

HEp-2 and Vero cells were from the stock of our institute and grown in Dulbecco's Minimum Essential Medium (DMEM) (Hyclone, USA) supplemented with 10% FBS (Thermo Fisher Scientific, USA) at 37 °C with 5% CO_2_. HSV-1 BAC Luc was a kind gift from Prof. Chunfu Zheng (Fujian Medical University, Fuzhou, China) [8]. Nrf2 expressing plasmid nHA-NFE2L2/pRK5 was constructed by Dr. Jiao Wang of our institute (unpublished). tBHQ (MedChemExpress, USA, CAS number: 1948–33-0) was diluted to the desired concentrations through a graded series of dilutions in dimethyl sulfoxide (DMSO) (SIGMA, USA). X-tremeGENE HP Transfection Reagent was purchased from Roche (Switzerland).

### Virus infection

HEp-2 cells were seeded at 4 × 10^5^ cells per well in 6-well plates (Corning, USA) and cultured overnight to investigate Nrf2 regulation during the process of HSV-1 infection. Cells were infected with HSV-1 at an multiplicities of infection (MOI) of 1. Samples were collected at 6, 12 and 24 h post-infection (hpi). 8-hydroxy-2'-deoxyguanosine (8-OHdG) assay was performed to monitor the level of oxidative stress induced by HSV-1 infection. Western Blot was performed to identify the expression of Nrf2 (Abcam, USA), Keap1 (Abcam, USA), HO-1 (CST, USA), NQO1 (CST, USA) and virus protein ICP4 (Abcam, USA). To further investigate the effect of virus infection on Nrf2-ARE pathway. Cells were infected with HSV-1 at an MOI of 0.5, 1, 2, 5, respectively. Western Blot was performed to identify the expression of Nrf2 and its regulated antioxidant enzymes.

To investigate the effect of Nrf2 upregulation on HSV-1 proliferation, HEp-2 cells were seeded at 2 × 10^5^ cells per well in 6-well plates (Corning, USA) and cultured overnight. Cells were pretreated with two different concentrations (1 µg, 0.5 µg) of Nrf2 plasmid 24 h before HSV-1 (MOI = 1) infection, then cultured in maintenance medium for 24 h, protein and total RNA were extracted. As mentioned above, Western Blot was performed to identify proteins' expression, including HSV-1 glycoprotein D (gD) (Abcam, USA). After being frozen and thawed for 3 times, the viral titer of the supernatant was measured by TCID_50_ assay. Real-Time PCR was performed to identify the expression of the interferon α (IFN-α), 2′-5′-oligoadenylate synthetase 1 (OAS1) and human ubiquitin-like modifier (ISG15).

To further explore the effect of Nrf2 endogenous activation on HSV-1 proliferation, HEp-2 cells were seeded at 2 × 10^5^ cells per well in 6-well plates (Corning, USA) and cultured overnight. Cells were pretreated with two different concentrations (10 µM, 25 µM) of tBHQ 12 h before HSV-1 (MOI = 1) infection, then cultured in a maintenance medium for 24 h. As mentioned above, Western Blot and Real-Time PCR were performed to identify the expression of protein and genes, respectively, and the viral titer was measured by TCID_50_ assay.

To further verify the protective role of Nrf2 against HSV-1, Nrf2 was knocked down by siRNA transfection. HEp-2 cells were seeded at 2 × 10^5^ cells per well in 6-well plates (Corning, USA) and cultured overnight. Cells were transfected with Nrf2-specific siRNA (100 nM) (Santa Cruz, USA) or nonspecific (NC) siRNA 12 h before HSV-1 (MOI = 1) infection. As mentioned above, Western Blot and Real-Time PCR were performed to identify the expression of protein and genes, respectively, and the viral titer was measured by TCID_50_ assay.

### Western blot analysis

The expression of proteins was measured by Western Blot according to the protocol described previously [9]. Briefly, cells were lysed using a lysis buffer containing 1 × Cocktail (SIGMA, USA). After centrifugation 12,000* g* at 4 °C for 15 min, equivalent amounts of protein samples were separated using 4–20% SDS-PAGE (Beyotime, China) and transferred onto a PVDF membrane (Millipore, USA) using the Semi-dry Transfer System (Bio-Rad, USA), then blocked with 5% skimmed milk at room temperature for 1 h, the corresponding primary antibody Nrf2, Keap1, HO-1, NQO1, ICP4, gD, and β-actin were successively added to the membrane and incubated at 4 °C overnight. Anti-rabbit IgG, HRP-linked antibody, and anti-mouse IgG, HRP-linked antibody (Abcam, USA) were used as secondary antibodies. The protein bands were detected using the enhanced chemiluminescence (ECL) Western blotting kit (Millipore, USA) and a Clinx ChemiScope imaging system (Clinx Science Instruments, China).

### Real-time PCR

Total RNA was extracted using the RNeasy Mini Kit (QIAGEN). A 1 μg aliquot of total RNA was used for reverse transcription reaction using the Evo M-MLV RT Kit with gDNA Clean for qPCR (Accurate Biotechnology Co. Ltd., China). Real-Time PCR was performed using a Gentier 96E system (TIANLONG Co. Ltd., China) with SYBR Green Premix Pro Taq HS qPCR Kit (Accurate Biotechnology Co. Ltd., China) according to the manufacturer's instructions. All samples were run in triplicate, and the relative gene expression was analyzed using 2^−△△CT^ method [10]. Primer were designed as described previously [11]. IFN-α (forward), 5′-AGAGTCACCCATCTCAGCAAG-3′, IFN-α (reverse), 5′-CACCAGGACCATCAGTAAAGC-3′; OAS1 (forward), 5′-TCAGTCAGCAGAAGAGATAA-3′, OAS1 (reverse), 5′-CAATGAACTTGTCCAGAGATT-3′; ISG15 (forward), 5′-GCAGATCACCCAGAAGAT-3′, ISG15 (reverse), 5′-CCTTGTTATTCCTCACCAG-3′, GAPDH (forward), 5′-GACACCCACTCCTCCACCTTT-3′, GAPDH (reverse), 5′-ACCACCCTGTTGCTGTAGCC-3′. Data were normalized to the expression of GAPDH. The experiment was performed in triplicate, and the results are presented as mean ± SDs; *P* < 0.05 indicated a significant difference.

### Virus titration

Vero cells were seeded at 1 × 10^4^ per well in 96-well plates (Corning, USA) and cultured overnight. A series of dilutions at 1:10 of the original virus samples were made, then transferred 100 μL of the diluted virus to 96-well plates, six parallel wells per dilution, and incubated in a 37 °C incubator with a 5% CO_2_ for 3–4 days. For each dilution, the number of wells that were positive for cytopathic effect (CPE) was scored. The viral titer was calculated by 50% Tissue Culture Infectious Dose (TCID_50_) following the Reed-Muench method [12]. The experiment was performed in triplicate, and the results are presented as mean ± SDs; *P* < 0.05 indicated a significant difference.

### Measurement of oxidative stress

The levels of 8-hydroxy-2'-deoxyguanosine (8-OHdG), as a measure of oxidative stress, was monitored using an 8-OHdG ELISA kits (mlbio Co. Ltd China) according to the manufacturer's instructions.

## Results

### HSV-1 infection induced oxidative stress and Nrf2 was activated in the early stage of HSV-1 infection

**Fig. 1:**
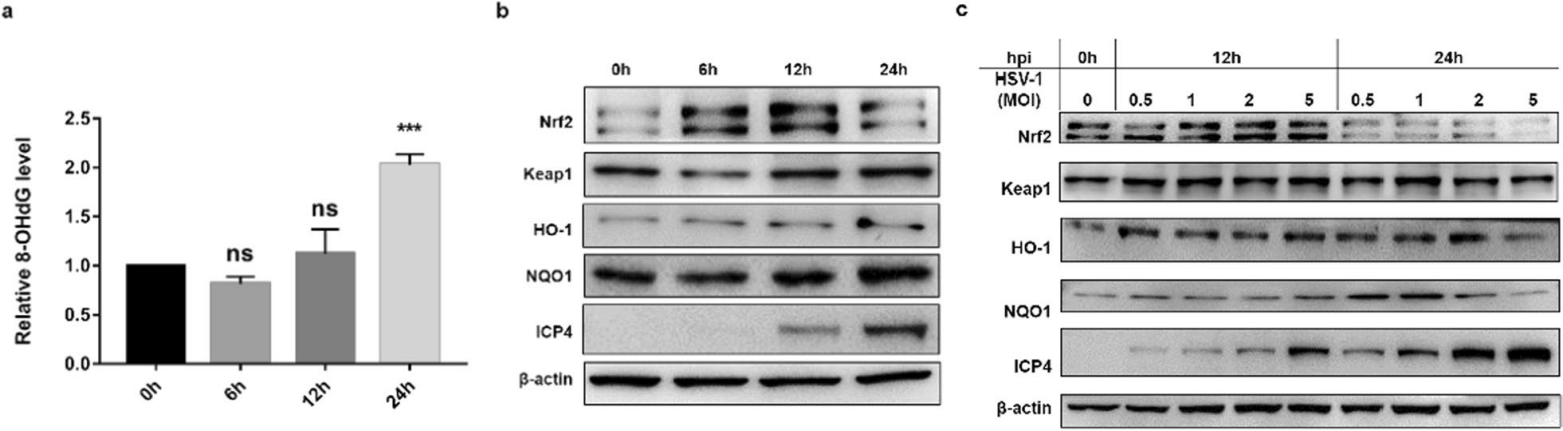
HSV-1 infection induced oxidative stress and Nrf2 was activated in the early stage of HSV-1 infection. **a** HEp-2 cells were infected with HSV-1 at an MOI of 1 and samples were collected to identify the oxidative stress by 8-OHdG ELISA kit at 0, 6, 12 and 24 hpi. **b** The expression of Nrf2, Keap1, HO-1, NQO1 and virus protein ICP4 were identified by Western Blot. **c** The expression of Nrf2-ARE pathway protein was detected after infected with HSV-1 at different MOIs by Western Blot. Data represent the results from three independent experiments (Mean ± SD, ***P < 0.001, ns, not significant)

To determine whether oxidative stress was induced by HSV-1 infection, the levels of 8-OHdG, a measure of oxidative stress, was monitored after HSV-1 infection at an MOI of 1. As shown in Fig. 1a, the relative 8-OHdG level was increased 24 h after infection with HSV-1 compared with uninfected HEp-2 cells. This result suggests that HSV-1 infection induced oxidative stress.

Since Nrf2-ARE pathway played an essential role in the regulation of antioxidant reaction, we detected the protein levels of HO-1 and NQO1, the canonical down-stream targets regulated by Nrf2 signaling. As shown in Fig. 1b, with the progress of HSV-1 infection, the expression of viral proteins of ICP4 (an immediate early proteins) gradually increased. Virus infection resulted in a increase in Nrf2 expression as early as 6 and 12 hpi. However, it returned to the basic level at 24 hpi. The antioxidant enzymes HO-1 and NQO1 were increased in a time-dependent manner.

To confirm that HSV-1 induced turnover of Nrf2, HEp-2 cells were infected with HSV-1 at different multiplicities of infection (MOI of 0.5, 1, 2 and 5), a dose-dependent reduction in Nrf2 protein levels was observed in infected cells at 24 hpi, supporting the idea that HSV-1 specifically interfered with Nrf2 expression. Particularly at 24 hpi, Nrf2 protein expression was reduced to a barely detectable level, and the expression of HO-1 and NQO1 decreased significantly after infection with HSV-1 at an MOI of 5.

The above results suggest that HSV-1 infection induced oxidative stress, Nrf2 and its down-stream antioxidant enzymes were activated during the early stage of HSV-1 infection (12 hpi), however, suppressed at alater stage (24 hpi).

### Overexpression of Nrf2 dampened HSV-1 replication

**Fig. 2:**
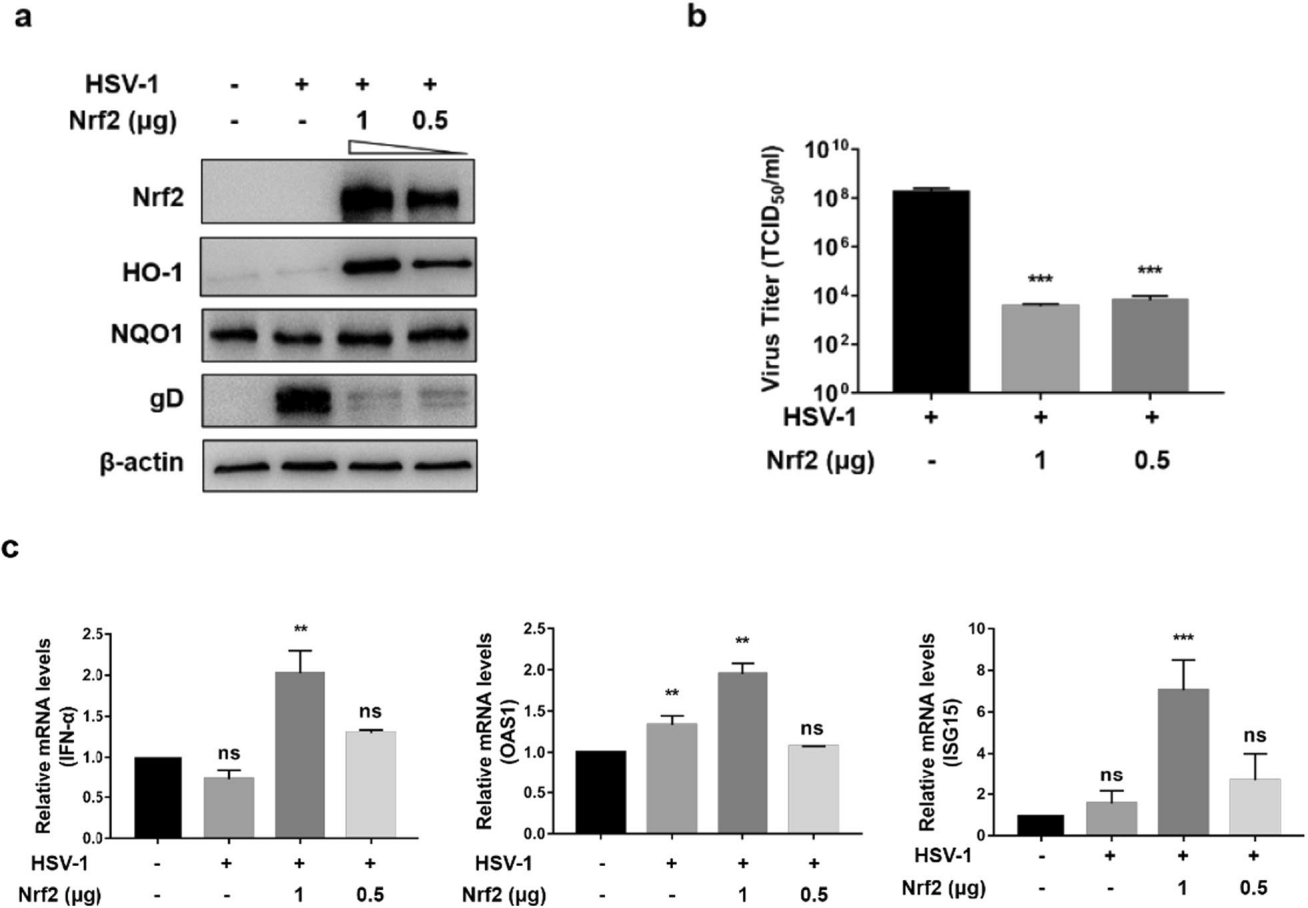
Ectopic expression of Nrf2 dampened HSV-1 replication. HEp-2 cells were pretreated with two different concentrations (1, 0.5 µg) of Nrf2 plasmid 24 h before HSV-1 (MOI = 1) infection, HEp-2 cells infected HSV-1 were used as positive control and untreated HEp-2 cells were used as negative control. Cells were cultured in maintenance medium for 24 h, then protein and total RNA were extracted. **a** The expression of Nrf2, HO-1, NQO1 and virus protein gD were identified by Western Blot. **b** Cells were repeatedly frozen and thawed for 3 times, then the viral titer of the supernatant was measured by TCID_50_ assay. **c** The mRNA level of IFN-α, OAS1 and ISG15 were detected by Real-Time PCR. Data represent the results from three independent experiments (Mean ± SD, **P < 0.01, ***P < 0.001, ns, not significant)

To investigate the effect of Nrf2 upregulation on HSV-1 proliferation. HEp-2 cells were pretreated with 1 µg or 0.5 µg of Nrf2 plasmid for 24 h before HSV-1 (MOI = 1) infection as described in the materials and methods. Western Blot showed that the expression of Nrf2 and its down-stream HO-1, NQO1 increased compared with untreated HEp-2 cells, while the virus protein gD decreased compared with HSV-1 infected cells after overexpression of Nrf2 (Fig. 2a). In addition, the virus titer was dramatically decreased, approximately from 1 × 10^8^ to 1 × 10^4^ TCID_50_/ml (Fig. 2b).

HO-1 has been known to regulate IFN production and play an important role in suppressing viral replications [13]. To determine whether HO-1 upregulation activates antiviral IFN response in the case of HSV-1 infection, the intracellular level of anti-inflammatory factors such as IFN-α, OAS1 and ISG15 was measured. As shown in Fig. 2c, overexpression of Nrf2 in HSV-1 infected cell enhanced the mRNA expressions of IFN-α, as well as protein expressions of some ISGs, such as OAS1 and ISG15. These results suggest that Nrf2 plays a role in controlling HSV-1 replication through HO-1 mediated IFN response.

### Nrf2 agonist tBHQ inhibit HSV-1 replication

To further explore the effect of Nrf2 endogenous activation on HSV-1 proliferation. The Nrf2-activating agonist, tBHQ, was used to assess the effect of Nrf2 activation on HSV-1 replication. tBHQ modifies cysteine residues in Keap1 through the formation of carbamodithioate and release Nrf2 from Nrf2-Keap1 complex, which results in the Nrf2 translocation into the nucleus [5]. Accordingly, HEp-2 cells were pretreated with 10 or 25 μM tBHQ for 12 h before HSV-1 (MOI = 1) infection as described in the materials and methods.

**Fig. 3:**
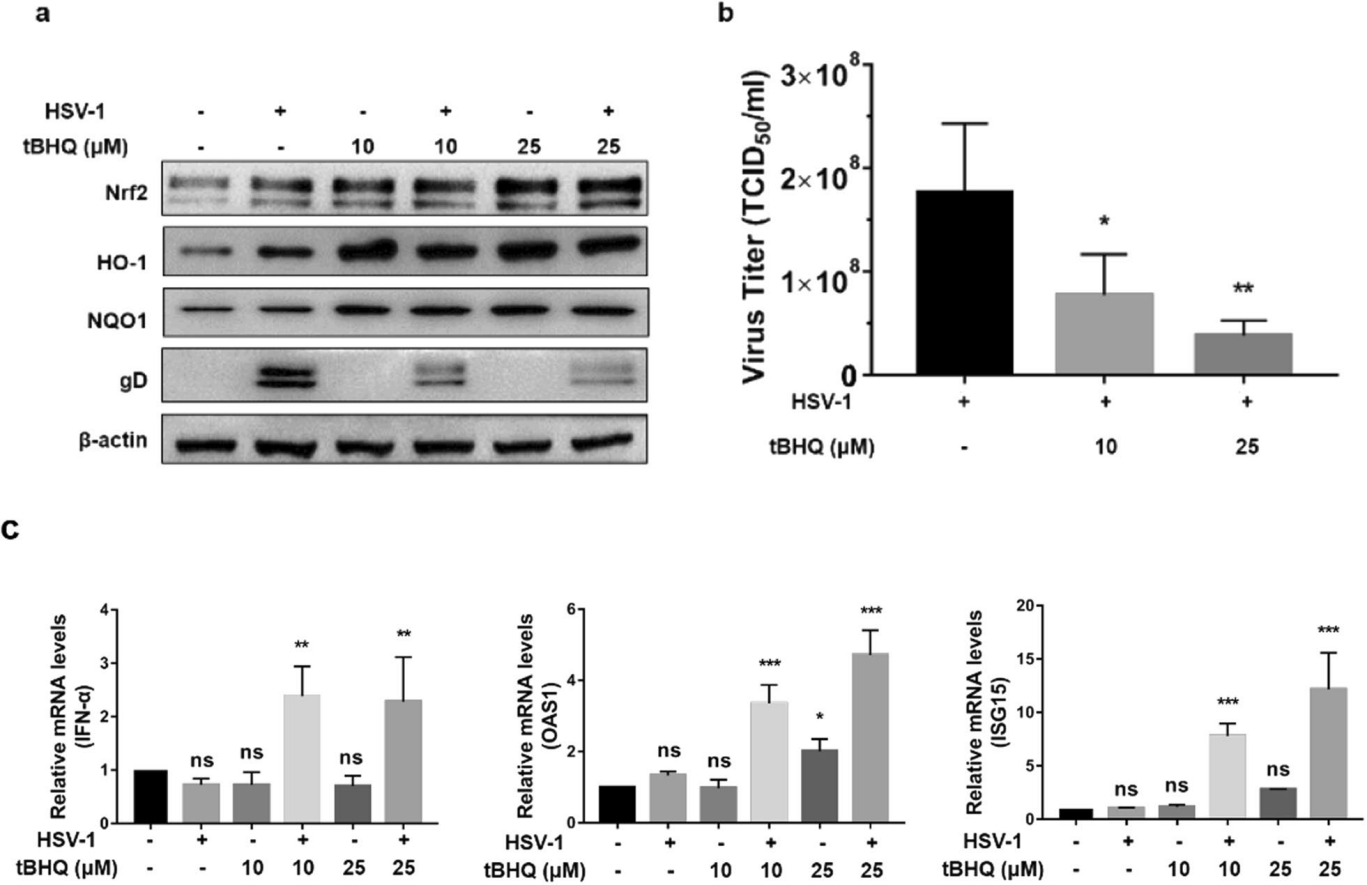
Nrf2 agonist tBHQ inhibit HSV-1 replication. HEp-2 cells were pretreated with two different concentrations (10 µM, 25 µM) of tBHQ 12 h before HSV-1 (MOI = 1) infection, HEp-2 cells infected HSV-1 were used as positive control and untreated HEp-2 cells were used as negative control. Cells were cultured in maintenance medium for 24 h, then protein and total RNA were extracted. **a** The expression of Nrf2, HO-1, NQO1 and virus protein gD were identified by Western Blot. **b** Cells were repeatedly frozen and thawed for 3 times, then the viral titer of the supernatant was measured by TCID_50_ assay. **c** The mRNA level of IFN-α, OAS1 and ISG15 were detected by Real-Time PCR. Data represent the results from three independent experiments (Mean ± SD, *P < 0.05, **P < 0.01, ***P < 0.001, ns, not significant)

As shown in Fig. 3a, Nrf2, HO-1 and NQO1 increased compared with untreated HEp-2 cells, while the virus protein gD decreased compared with HSV-1 infected cells pretreated with tBHQ. In addition, the virus titer was decreased about 5.0 to 10.0-fold. Activation of Nrf2 in HSV-1 infected cells enhanced the mRNA expressions of IFN-α, as well as protein expressions of some ISGs, such as OAS1 and ISG15 (Fig. 3c).

Altogether, these results suggest that upregulation of Nrf2 or activation of endogenous Nrf2 promotes the expression of down-stream antioxidant enzymes, and Nrf2-ARE pathway controlled the basal expression of these inflammatory genes, which effectively inhibit the proliferation of HSV-1.

### Silencing of Nrf2 promotes virus replication

**Fig. 4:**
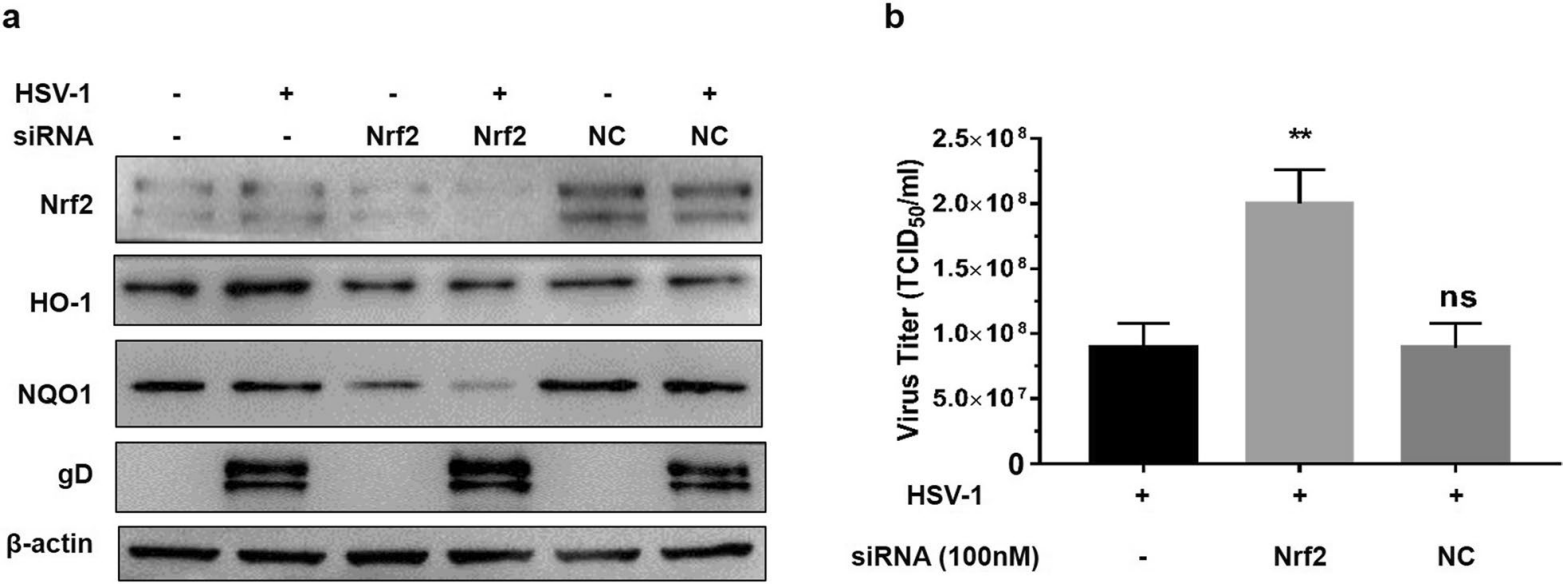
Silencing of Nrf2 promotes virus replication. HEp-2 cells were pretreated with Nrf2 siRNA (100 nM) or NC siRNA (100 nM) 12 h before HSV-1 (MOI = 1) infection, HEp-2 cells infected HSV-1 were used as positive control and untreated HEp-2 cells were used as negative control. Cells were cultured in maintenance medium for 24 h, then protein and total RNA were extracted. **a** The expression of Nrf2, HO-1, NQO1 and virus protein gD were identified by Western Blot. **b** Cells were repeatedly frozen and thawed for 3 times, then the viral titer of the supernatant was measured by TCID_50_ assay. Data represent the results from three independent experiments (Mean ± SD, **P < 0.01, ns, not significant)

To further evaluate the protective role of Nrf2 against HSV-1, Nrf2 was knocked down by siRNA transfection. Compared with the untreated HEp-2 cells, transfected with Nrf2 siRNA decreased the expression of Nrf2 by up to 90%. Meanwhile, the protein levels of Nrf2 down-stream HO-1 and NQO1 were also significantly decreased (Fig. 4a). In terms of HSV-1 replication, knocking down the expression of Nrf2 increased the expression of the HSV-1 gD (Fig. 4a) and the virus titer (Fig. 4b) approximately by 2.0-fold, respectively. These data showed that knocking down the expression of Nrf2 effectively downregulated the cellular antioxidant ability, and Nrf2-ARE pathway plays a critical part in the defence against viral infection.

## Discussion

Oxidative stress has recently been determined to interfere with the infection of HSV-1 [14, 15]. The imbalance between ROS production and the antioxidant defensive system is involved in maintaining intracellular homeostasis and the viral infection process [2]. Nrf2 is one of the key transcriptional factors involved in coping with oxidative stress of antioxidants by regulating the down-stream cellular antioxidant response [16]. However, the role of Nrf2 in HSV-1 infection is still unclear. In this study, we investigated the interactions between Nrf2 and HSV-1 replication.

In this study, our data showed that (i) HSV-1 infection triggered oxidative stress. (ii) Nrf2 was activated during the early stage of HSV-1 infection (12 hpi). (iii) Upregulation of Nrf2 inhibits HSV-1 replication while silencing of Nrf2 promotes viral infection. (iv) Nrf2-ARE pathway regulates the expression of inflammatory genes and the proliferation of HSV-1.

We found that the upregulation of Nrf2 in a transient manner after HSV-1 infection (Fig. 1b and 1c), which was also confirmed in Kaposi's Sarcoma-Associated Herpesvirus (KSHV) and Dengue Virus infection [17, 18]. It has been reported that Nrf2 degradation caused by viral infection is regulated by Keap1-dependent or Keap1-independent ubiquitin–proteasome pathways [19]. Bovine herpesvirus type infection stimulates Nrf2 degradation through the Keap1-dependent ubiquitin proteasome pathway [20]. However, Dengue virus uses the NS2B3 protease complex to strategically target Nrf2 for degradation [18]. In our study, Nrf2 was inhibited at 24 hpi, at this time, Keap1 was upregulated only in the virus infection group with an MOI of 1, but no significant change in higher MOI group (Fig. 1c). Therefore, we speculated that the ubiquitination degradation of Nrf2 may be caused by multiple ways, and the specific mechanism needs to be further studied.

It has been reported that upregulation of Nrf2 by overexpression of Nrf2 or supplementation with Nrf2 activators could significantly suppress the replication of several viruses [21–23]. Upregulation of Nrf2 by tBHQ rendered mice less susceptible due to Murine Cytomegalovirus (MCMV) infection [22]. Consistent with this, overexpression of Nrf2 decreased virus replication, influenza A nucleoprotein expression, antiviral response, and oxidative stress [23]. In this paper, we found that HSV-1 propagation was regulated by Nrf2. Overexpression of Nrf2 followed by HSV-1 infection, the expression of viral proteins and the viral titer decreased dramatically (Fig. 2a and b), while pretreated cells with Nrf2 agonist before HSV-1 infection showed a slight suppression of HSV-1 replication (Fig. 3a and b). The reason being that the activation effect of Nrf2 may persist for a very short time after removing the exogenous agonist, and the expression of antioxidant enzymes activated by the agonist was not as high as that in the Nrf2 plasmid transfection group. On the contrary, silencing of Nrf2 promotes HSV-1 infection (Fig. 4a and b).

Recent studies have shown that Nrf2-ARE pathway is not only involved in the regulation of oxidative stress but also to regulate inflammatory and antiviral responses [24]. A previous study showed that HO-1, an antioxidant enzyme down-stream of Nrf2, has the ability to activate IFN-α/β that inhibits replication of influenza virus [13]. IFN-α has been demonstrated to be essential in defending HSV-1 infection by induction of IFN-stimulated genes (ISGs), which works in synergy to inhibit viral replication via multiple distinct mechanisms [25]. Here, we found that activation of Nrf2 up-regulated the expression of HO-1, which can activate the IFN response and induce the expression of IFN-stimulated genes, thereby leading to efficient anti-HSV-1 effects (Figs. 2c and 3c). Thus, the antiviral activities of Nrf2 activation are much likely to be associated with the induction of ISGs. ROS are required for the activation of NF-kB and IRF-3 pathways to induce the production of type I IFN in HSV-1 infected cells [14], and meanwhile Nrf2-ARE pathway regulates ROS production [2]. Therefore, the cross talk between Nrf2 mediated antioxidant response and antiviral response needs to be further investigated.

## Conclusion

Our study showed that Nrf2 was activated in the early stage of HSV-1 infection and involved in regulating antioxidant response. Upregulation of Nrf2 by overexpression or its agonist represses the replication of HSV-1. On the contrary, silencing of Nrf2 promotes virus replication. Taken together, the Nrf2-ARE pathway is not only involved in the regulation of oxidative stress but also in inflammatory regulation and antiviral responses upon HSV-1 infection, thus targeting the Nrf2 pathway has potential for combating HSV-1 infection.

## Data Availability

All data generated or analyzed during this study are included in this published article.

## Abbreviations

Nrf2: Nuclear factor E2-related factor 2
HSV-1: Herpes simplex virus type 1
ROS: Reactive oxygen species
HO-1: Heme oxygenase-1
NQO1: NAD (P) H quinone oxidoreductase 1
tBHQ: Tert-Butylhydroquinone
IFN-α: Interferon α
OAS1: 2',5'-Oligoadenylate synthetase 1
ISG15: Human ubiquitin-like modifier
CPE: Cytopathic effect
TCID_50_: 50% Tissue Culture Infectious Dose
